# An Avoidable Cognitive Error in Chest Radiography

**DOI:** 10.5334/jbsr.3353

**Published:** 2023-12-28

**Authors:** Laura Haddad, Hanna Salame, Denis Tack

**Affiliations:** 1 Université libre de Bruxelles, Belgium; 2 Department of Radiology, Epidura La Madeleine, Rue Maria Thomée, 1, 7800 Ath, Belgium

**Keywords:** interpretation error, chest radiography, missed foreign body, training, diagnostic accuracy

## Abstract

**Teaching Point:** Awareness in radiology reporting of cognitive errors such as the alliterative bias can help minimize the delay to diagnosis and accelerate adequate patient care.

## Case History

**Figure 1:**
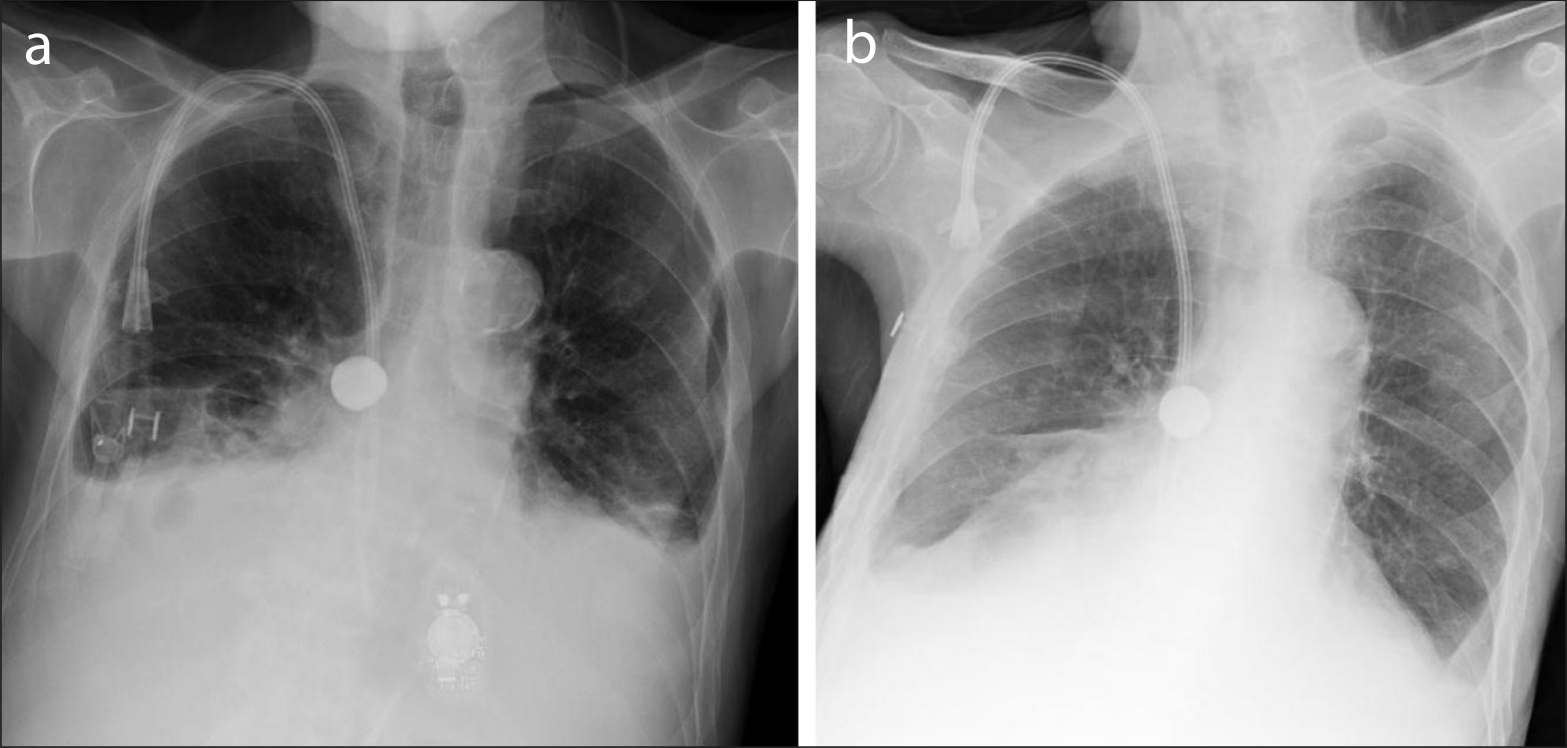


**Figure 2:**
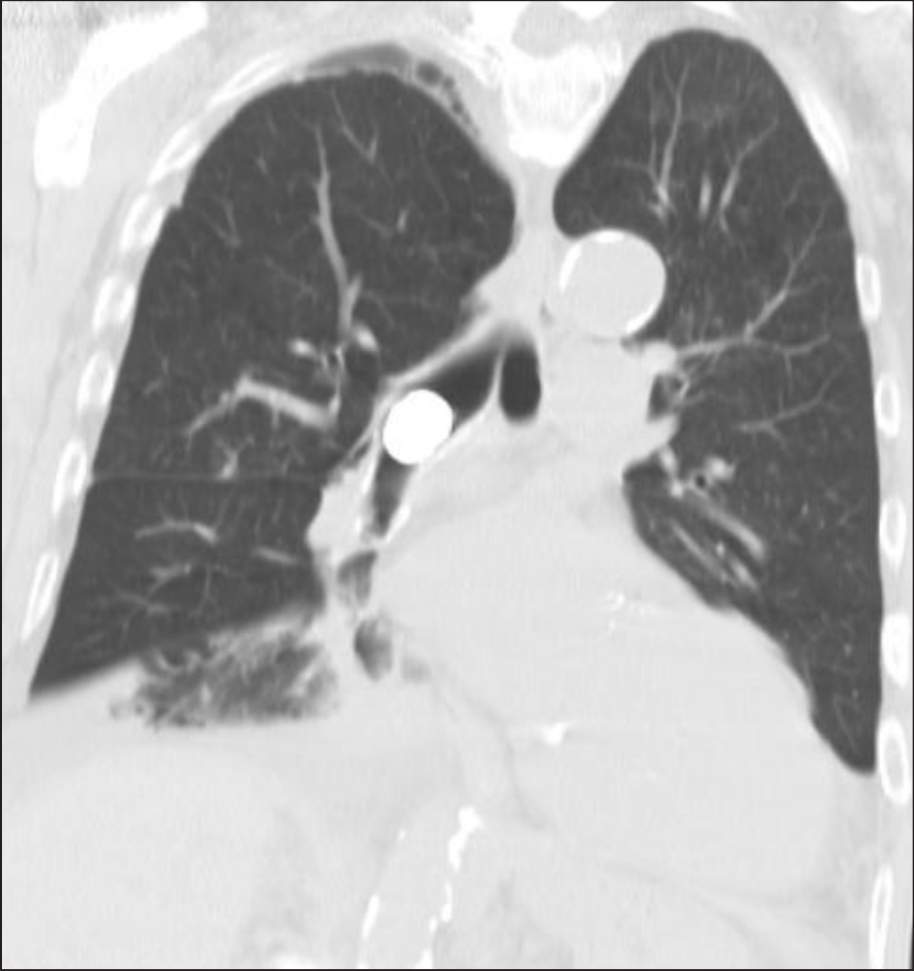


A 79-year-old male patient on hemodialysis for chronic renal failaure, presented with persistent cough and reccurrent pneumonias over the last few months. A chest radiograph (Figure 1a) showed blunting of both costophrenic angles as well as bibasilar infiltrates and atelectasis, predominant in the right lower lung field, and associated with bilateral volume loss. A hemodialysis catheter in adequate position and an electronic device overlying the heart were seen. A rounded opaque structure is noted at the level of the main right bronchus. This last element was not mentioned in the initial report by the radiologist. The clinician observed this and the radiologist reviewed all available images. This opaque structure was present on chest radiographs performed one month (not shown) and two months earlier (Figure 1b). Chest computed tomography (CT) (Figure 2) showed a round dense structure located within the right main bronchus, probably aspirated by the patient. A bronchospy was performed and a metal dental crown was removed.

## Comments

Most malpractice issues in radiology are related to diagsnotic errors [1]. The majority of these errors are perceptual errors when the radiologist fails to detect an abnormality. Less commonly, diagnostic errors are interpretative errors. A mental shortcut or a systemic error in reasoning are the main causes of diagnotic errors [1]. This case of successive chest radiographs with the same error draws the attention to the importance of awarness of the mechanisms of errors. This round structure is obviously clearly visible on the radiographs. Thus, it was probably seen, yet considered as not relevant, making it an interpretative error. If on the first radiograph it was a simple error of judgement, on the second and third radiograph, it is more likely to be related to the alliterative bias. This cognitive bias occurs when the radiologist is largely influenced by prior reports with a greater likelihood of repeating the same error [1]. This error could have been easily avoided if the radiologists were just aware or reminded of this specific interpretative cognitive bias known as the alliterative bias, as illustrated in this simple case.
